# Methylprednisolone effects on serum biochemical factors (CRP, PCT, IL-6, TNF-a) in viral pneumonia

**DOI:** 10.5937/jomb0-53124

**Published:** 2025-06-13

**Authors:** Hongping Zeng, Qi Zhu, Shaoyu Bai, Jie Liu, Dengjie Ren, Xin Chen

**Affiliations:** 1 Zigong First People's Hospital, Department of Traditional Chinese Medicine, Zigong, Sichuan Province, China

**Keywords:** methylprednisolone, azithromycin, viral pneumonia, dosage, biochemical factors, CRP, PCT, IL-6, TNF-a, metilprednizolon, azitromicin, virusna pneumonija, doziranje, biohemijski faktori, CRP, PCT, IL-6, TNF-a

## Abstract

**Background:**

This study was performed to demonstrate the effects of methylprednisolone (MPS) and azithromycin treatment on serum biochemical factors and their impact on Serum Biochemical Factors (CRP, PCT, IL-6, TNF-a) prognosis in patients with viral pneumonia (VP).

**Methods:**

This was a non-randomised clinical trial study on 120 patients with VP admitted to our hospital who had received different doses of methylprednisolone for viral pneumonia. Subjects were collected in four groups of Controls (Ctrl), low-dose (40 mg MPS, L-MPS), medium-dose (80 mg MPS, M-MPS), and high-dose (120 mg MPS, H-MPS) groups. The therapeutic efficacy of each group was evaluated. C-reactive protein (CRP), procalcitonin (PCT), and interleukin-6 (IL-6) were detected. Pulmonary function parameters were assessed using a pulmonary function testing device. Adverse reactions (ARs) such as fever, nausea, vomiting, and gastrointestinal bleeding post-treatment were recorded.

**Results:**

The total effective rate (TER) post-treatment in the Ctrl group was 60.00%, which was inferior to that in the L-MPS group (76.67%), M-MPS group (90.00%), and H-MPS group (93.33%) (P<0.05). Disappearance time of CRP, PCT, IL-6, TNF-a, fever, cough, and X-ray infiltrates was reduced in L-MPS, M-MPS, and H-MPS groups relative to the Ctrl group (P<0.05), while FVC, MMEF, and PEF were increased (P<0.05). Disappearance time of CRP, PCT, IL-6, TNF-a, fever, cough, and X-ray infiltrates in M-MPS and H-MPS groups was inferior to that in the L-MPS group (P<0.05), while FVC, MMEF, and PEF were higher (P<0.05).

**Conclusions:**

Medium-dose (80 mg) MPS combined with azithromycin greatly reduces inflammatory cytokine levels, shortens the time to clinical symptom relief, and improves lung function and respiratory capacity, demonstrating significant efficacy in treating VP.

## Abstract (sr)

**Uvod:**

Ova studija je sprovedena kako bi se prikazali efekti tretmana metilprednizolonom (MPS) i azitromicinom na serumske biohemijske faktore i njihov uticaj na prognozu serumskih biohemijskih faktora (CRP, PCT, IL-6, TNF-a) kod pacijenata sa virusnom pneumonijom (VP).

**Metode:**

Ovo je bila nerandomizovana klinička studija sprovedena na 120 pacijenata sa virusnom pneumonijom (VP) primljenih u našu bolnicu, i koji su primili različite doze metilprednizolona za lečenje virusne pneumonije. Ispitanici su rasporedeni u cetiri grupe: kontrolnu grupu (Ctrl), grupu sa niskom dozom (40 mg MPS, L-MPS), grupu sa srednjom dozom (80 mg MPS, M-MPS) i grupu sa visokom dozom (120 mg MPS, H-MPS). Procenjena je terapijska efikasnost svake grupe. Merene su vrednosti C-reaktivnog proteina (CRP), prokalcitonina (PCT) i interleukina-6 (IL-6). Parametri plućne funkcije su procenjeni pomću uređaja za testiranje funkcije pluća. Zabeležene su i neželjene reakcije nakon tretmana, kao što su groznica, mučnina, povraćanje i gastrointestinalno krvarenje.

**Rezultati:**

Ukupna stopa efikasnosti (TER) nakon tretmana u kontrolnoj grupi (Ctrl) je iznosila 60,00%, što je bilo niže u poređenju sa grupom L-MPS (76,67%), grupom M-MPS (90,00%) i grupom H-MPS (93,33%) (P<0,05). Vreme nestanka CRP-a, PCT-a, IL-6, TNF-a, groznice, kašlja i infiltrata na rendgenskom snimku je bilo smanjeno u grupama L-MPS, M-MPS i H-MPS u odnosu na kontrolnu grupu (P<0,05), dok su vrednosti FVC, MMEF i PEF bile povećane (P<0,05). Vreme nestanka CRP-a, PCT-a, IL-6, TNF-a, groznice, kašlja i infiltrata na rendgenskom snimku u grupama M-MPS i H-MPS je bilo kraće u odnosu na grupu L-MPS (P<0,05), dok su vrednosti FVC, MMEF i PEF bile veće (P<0,05).

**Zaključak:**

Srednja doza (80 mg) MPS u kombinaciji sa azitromicinom značajno smanjuje nivoe inflamatornih citokina, skraćuje vreme za ublažavanje kliničkih simptoma i poboljšava funkciju pluća i respiratorni kapacitet, pokazujući značajnu efikasnost u lečenju virusne pneumonije.

## Introduction

Viral pneumonia (VP) is a respiratory tract infection caused by various viruses, with common pathogens of influenza, respiratory syncytial, etc. Upon entry into the human body, viruses spread downwards to the lungs, triggering inflammatory reactions in the alveoli and bronchi [1]. VP typically occurs in immunocompromised populations, such as the elderly, children, and individuals with chronic diseases or compromised immune systems [2]. Symptoms during the onset of the disease include fever, headache, generalised body aches, dry cough, and pulmonary infiltrates. As the disease progresses, lung involvement increases, impairing lung function [3]. Current treatment methods for VP primarily include antiviral therapy, supportive care, anti-inflammatory treatment, and immunomodulatory therapy. Antiviral drugs can directly target specific pathogens, inhibiting viral replication, and represent an important approach in treating VP.

Nevertheless, the use of antiviral drugs may lead to the development of drug resistance [4]. Supportive care involves ensuring adequate rest, maintaining fluid and electrolyte balance, and providing respiratory support, which helps stabilise patients’ vital signs. While supportive care is simple and effective, it does not eradicate the pathogen [5]. Immunomodulatory therapy aims to enhance the patient’s immune response against the virus and promote recovery. Nevertheless, the effectiveness of immunomodulatory therapy varies among individuals, and its mechanisms are complex [6]. Anti-inflammatory treatment aims to reduce the body’s inflammatory response, alleviate pulmonary symptoms, and relieve the patient’s condition, although it may still entail certain side effects [7]. Therefore, seeking effective and safe treatment strategies is crucial for improving VP patients’ treatment outcomes and prognosis.

Azithromycin is a broad-spectrum antibiotic commonly adopted to treat respiratory tract infections and skin soft tissue infections. Its application in treating VP primarily involves treating secondary bacterial infections and preventing bacterial complications. Research suggests that azithromycin may help inhibit viral replication and possesses anti-inflammatory and immunomodulatory effects [8]. Methylprednisolone (MPS), a synthetic corticosteroid hormone, is commonly utilised for anti-inflammatory and anti-allergic therapy. In patients with VP, MPS is primarily utilised to treat excessive inflammation, such as pulmonary inflammation and cytokine release syndrome seen in severe cases [9]. MPS can suppress inflammation, attenuate excessive immune system activation, and thereby alleviate tissue damage [10]. Nevertheless, prolonged use of corticosteroids may cause immune suppression and high infection risk [11] [12].

MPS and azithromycin, commonly applied medications for treating VP, may have significant implications for patient prognosis when used in combination at different doses. Nevertheless, current research on the impact of this combination therapy on serum biochemical factors and prognosis in patients with VP remains relatively limited. This work investigated the effects of different doses of MPS combined with azithromycin on serum biochemical factors and prognosis in patients with VP. By examining the influence of this combination therapy on serum biochemical factors and understanding its impact on the prognosis of VP, a deeper understanding of the therapeutic effects of this regimen on the pathophysiological processes of VP can be obtained, thus providing theoretical support for future treatment strategies.

## Materials and methods

### Subjects

This was a non-randomised prospective clinical trial study. Between January 2021 and December2023, our hospital admitted 120 patients diagnosed with VP. Consecutive sampling was used to select patients for the study. Consecutive patients who met the inclusion and exclusion criteria were approached for participation in the study. The physician in charge, a board-certified pulmonologist, assigned patients to one of the four groups based on the severity of their VP symptoms and medical history, as below: control (Ctrl), low-dose MPS (L-MPS), medium-dose MPS (M-MPS), and high-dose MPS (H-MPS), with 30 patients in each group.

The sample size for each group was determined based on a priori power analysis to ensure adequate statistical power to detect significant differences between groups, assuming a moderate effect size (Cohen’s d=0.5) and a two-tailed alpha level of 0.05. The power analysis indicated that a sample size of 30 patients per group would provide 80% power to detect significant differences between groups.

### Inclusion criteria

Patients were eligible for this study if they were diagnosed with viral pneumonia (VP) and met the following criteria: (1) aged 18 years or older; (2) presented with symptoms of VP, including cough, fever, and dyspnea; (3) had a confirmed diagnosis of VP based on clinical evaluation, laboratory tests (e.g., PCR, serology), and imaging studies (e.g., chest X-ray, CT scan); (4) had no underlying chronic respiratory diseases, such as chronic obstructive pulmonary disease (COPD) or asthma; (5) had no immunocompromised status, including HIV/AIDS, cancer, or taking immunosuppressive medications; (6) had no prior history of VP or other severe respiratory infections within the past 6 months; and (7) were able to provide informed consent. Patients were excluded if they had any of the following: (1) bacterial pneumonia or other bacterial infections; (2) severe comorbidities, such as heart failure, liver cirrhosis, or renal failure; (3) were pregnant or breastfeeding; or (4) were unable to complete the study due to language barriers or cognitive impairment.

### Treatment regimens

All patients received basic treatment with oseltamivir phosphate capsules, budesonide, piper - acillin sodium and tazobactam sodium. The basic treatment regimen was as follows: OseltamivirPhosphate Capsules (Jiangsu Sinopep-Allsino Biopharmaceutical Co., Ltd; National Medical Products Administration (NMPA) number H20212249; specification: 75 mg×10 granule) was administered for expectoration, twices daily. Concurrently, nebulised inhalation of budesonide (CF PharmTech, Inc.; NMPA NO. H20213357; specification: 2 mL: 1 mg) was performed, along with Piperacillin Sodium and Tazobactam Sodium (China meheco sanyang pharmaceutical Co., Ltd.; NMPA NO. H20073584; specification: 4.5 g) for intravenous injection with 4.5 g once every 8 h.

For the Ctrl group, in addition to basic treatment, azithromycin (Zhejiang Huarun three nine public benefit pharmaceutical Co., Ltd.; NMPA No. H20084458; specification: 0.25 g × 6 pill), take 0.5 g orally for the first time, then maintain with 0.25 g once daily for 7 days.

For the L-MPS group, low-dose MPS (Pfizer Inc., Belgium; NMPA No.HJ20170197; specification: 40 mg/btl) was combined with azithromycin for treatment. The dosage of azithromycin was the same as that in the Ctrl group. MPS was administered at 40 mg/day, added to 0.9% NaCl injection at a ratio of 1 mg/2.5 mL, and intravenously for 7 days.

For the M-MPS group, medium-dose MPS was combined with azithromycin for treat ment. The dosage of azithromycin was the same as that in the Ctrl group. MPS was administered at 80 mg/day, added to 0.9% NaCl injection at a ratio of 1 mg/2.5 mL, and intravenously for 7 days.

For the H-MPS group, hgih-dose MPS was combined with azithromycin for treat ment. The dosage of azithromycin was the same as that in the Ctrl group. MPS was administered at 120 mg/day, added to 0.9% NaCl injection at a ratio of 1 mg/2.5 mL, and intravenously for 7 days.

### Observation indicators

The efficacy of treatments in different groups was evaluated based on the time taken to relieve clinical symptoms and findings from pulmonary imaging examinations. Response criteria were defined as follows: Marked improvement: basic symptoms mostly disappeared, and X-ray examination showed disappearance of pulmonary shadows; Effective: basic symptoms significantly improved compared to pretreatment, and X-ray examination showed a reductionof pulmonary shadows; Ineffective: clinical symptoms showed no improvement or worsened compared to pre-treatment, and the area of pulmonary shadows increased. The total effective rate (TER) of treatment was calculated and compared among the groups.

Before and post-treatment, 3 mL fasting venous blood samples were collected from the patients’ elbows. After centrifugation (3,000 rpm, 15 min), serum was separated and used for enzyme-linked immunosorbent assay to detect C-reactive protein (CRP), procalcitonin (PCT), and interleukin-6 (IL-6). Tumor necrosis factor-alpha (TNF-α) levels were measured using a chemiluminescent immunometric assay.

Patients were seated, and lung function was assessed using a pulmonary function testing device (Masteren Difunsion, Degeer GmbH, Germany). Pulmonary function parameters, including forced vital capacity (FVC), maximal mid-expiratory flow (MMEF), and peak expiratory flow (PEF), were collected.

The occurrence of adverse reactions (ARs) such as fever, nausea, vomiting, and gastrointestinal bleeding post-treatment was statistically analysed for both groups [13].

### Statistical processing

The experimental data were processed using SPSS 19.0. For metric data conformed to a normaldistribution, means ± standard deviations (±s) were used for representation. Independent sample *t*-tests were employed for pairwise comparisons of metric data between groups. An analysis of variance (ANOVA) was conducted to compare multiple groups with dose data. Count data were denoted as percentages (%), and a chi-square test was utilised, with *P*<0.05 considered statistically significant.

## Results

In the Ctrl group, there were 18 males and 12 females, aged 19 to 68 (averaging 44.66) years, with a mean fever duration of 19.16 hours. The L-MPS group included 17 males and 13 females, aged 20 to 68 (averaging 45.03) years, with a mean fever duration of 19.33 hours. The M-MPS group had 18 males and 12 females aged 19 to 69 (averaging 44.84) years, with a mean fever duration of 18.95 hours. In the H-MPS group, there were 16 males and 14 females aged 20 to 67 (averaging 45.20) years, with a mean fever duration of 19.46 hours. Gender ratio, age, and baseline data, including average fever duration, were similar across all groups (*P*>0.05), indicating comparability.

The distribution of patients with marked improvement, effectiveness, and ineffectiveness posttreatment is shown in Table 1. The number of patients with marked improvement in the Ctrl group was inferior to that in the three dose groups (*P*<0.05), and that in the L-MPS group was inferior to that in other dose groups (*P*<0.05). The number of patients with marked improvement differed slightly between M-MPS and H-MPS groups (*P*>0.05). The number of patients with ineffectiveness in the Ctrl group was superior to that in the three dose groups (*P*<0.05). The number of patients with ineffectiveness in the L-MPS group was superior to that in other dose groups (*P*<0.05). The number of patients with ineffectiveness differed inconsiderably between M-MPS and H-MPS groups (*P*>0.05). The TER post-treatment in the Ctrl group was 60.00%, which was inferior to that in the three dose groups (76.67%, 90.00%, and 93.33%, respectively) (*P*<0.05). That in the L-MPS group was inferior to that in other dose groups (*P*<0.05), while the TER demonstrated neglectable differences between the M-MPS and H-MPS groups (*P*>0.05).

**Table 1:** Demographic characteristics. M = Male, F = Female

Variable	Ctrl	L-MPS	M-MPS	H-MPS	p-value
Gender	18M (60%),<br>12F (40%)	17M (57%),<br>13F (43%)	18M (60%),<br>12F (40%)	16M (53%),<br>14F (47%)	0.514
Age (years)	19–68<br>(44.66±10.2)	20–68<br>(45.03±11.5)	19–69<br>(44.84±9.8)	20–67<br>(45.20±12.1)	0.074
Mean Fever Duration<br>(hours)	19.16±2.5	19.33±2.8	18.95±2.2	19.46±3.1	0.126

Results showed that all three doses of MPS (L-MPS, M-MPS, and H-MPS) significantly reduced CRP, PCT, IL-6, and TNF-α levels compared to the control group (*P*<0.001). However, the L-MPS group showed superior reductions in these markers compared to the M-MPS and H-MPS groups (*P*<0.01), while there were no significant differences between the M-MPS and H-MPS groups (*P*>0.05).

**Figure 1:**
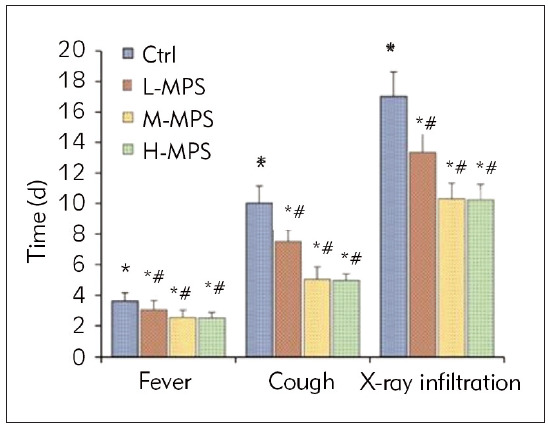
Comparison of time to resolution of clinical symptoms among different treatment regimens. Note: *P<0.05 vs. Ctrl; #P<0.05 vs. L-MPS

Figure 1 shows that the disappearance time of fever, cough, and X-ray infiltrates in the three dosegroups was shorter than that in the Ctrl group (*P*<0.05) and that in the L-MPS group was longerthan that in other dose groups (*P*<0.05). Nevertheless, the disappearance time of fever, cough, and X-ray infiltrates between M-MPS and H-MPS groups exhibited no marked differences (*P*>0.05).

The post-treatment pulmonary function statistics of all groups are presented in Table 4. FVC, MMEF, and PEF values in the three dose groups were superior to those in the Ctrl group (*P*<0.05). Additionally, the FVC, MMEF, and PEF values in the L-MPS group were inferior to those in other dose groups (*P*<0.05). Nevertheless, there was neglectable difference in the FVC, MMEF, and PEF values between M-MPS and H-MPS groups (*P*>0.05). Table 2 Table 3

**Table 2:** Treatment efficacy among different treatment regimens. Note: versus Ctrl, * indicates P<0.05; versus L-MPS, # indicates P<0.05.

Group	Case	Marked improvement	Effectiveness	Ineffectiveness
Ctrl	30	9 (30.00)	9 (30.00)	12 (40.00)
L-MPS	30	13 (43.33)*	10 (33.33)	7 (23.33)*
M-MPS	30	16 (53.33)*#	13 (36.67)	3 (10.00)*#
H-MPS	30	16 (53.33)*#	12 (40.00)	2 (6.67)*#

**Table 3:** Serum levels of inflammatory markers before and post-treatment.

Variable	Ctrl	H-MPS	M-MPS	L-MPS
Baseline CRP (mg/L)	2.5±0.5	3.1±0.7	3.4±0.8	2.8±0.6
Post-treatment CRP (mg/L)	1.2±0.3	1.5±0.4	2.1±0.6	1.8±0.5
P-value (ΔCRP)	<0.001	<0.001	<0.001	<0.001
Baseline PCT (ng/mL)	0.5±0.1	0.7±0.2	0.8±0.2	0.6±0.1
Post-treatment PCT (ng/mL)	0.2±0.1	0.3±0.1	0.5±0.1	0.4±0.1
P-value (ΔPCT)	<0.001	<0.001	<0.001	<0.001
Baseline IL-6 (pg/mL)	10.2±2.1	14.5±3.5	16.8±3.8	12.1±2.5
Post-treatment IL-6 (pg/mL)	5.5±1.2	7.2±1.8	8.9±2.1	6.5±1.5
P-value (ΔIL-6)	<0.001	<0.001	<0.001	<0.001
Baseline TNF-α (pg/mL)	3.5±0.8	5.2±1.2	6.1±1.4	4.2±1.0
Post-treatment TNF-a (pg/mL)	4.219±0.638	10.985±3.219	8.985±2.387	6.821±1.819
P-value (ΔTNF-α)	<0.001	<0.001	<0.001	<0.001
P-value (L-MPS vs M-MPS & H-MPS)	>0.05	<0.01	>0.05	>0.05
P-value (M-MPS vs H-MPS)	>0.05		>0.05	>0.05

**Table 4:** Pulmonary function among different treatment regimens. Note: ^*^P<0.05 vs. Ctrl; ^#^P<0.05 vs. L-MPS.

Group	FVC (L)	MMEF (L/s)	PEF (L/s)
Ctrl	2.02±0.35	1.86±0.49	5.42±1.05
L-MPS	2.21±0.47^*^	2.18±0.48^*^	6.61±1.21^*^
M-MPS	2.64±0.29^*#^	2.88±0.67^*#^	7.84±1.03^*#^
H-MPS	2.70±0.44^*#^	2.95±0.56^*#^	7.93±0.54^*#^

**Figure 2:**
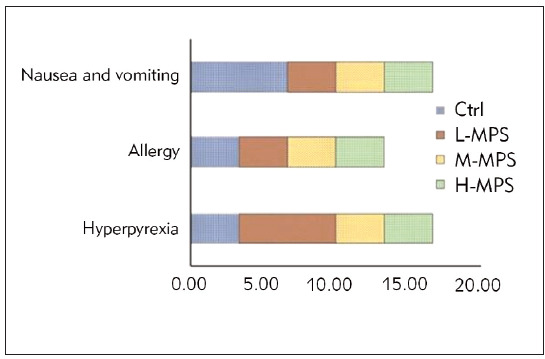
Comparison of ARs among different treatment regimens.

The statistical analysis of ARs, such as high fever, allergy, and nausea/vomiting post-treatment in all groups is depicted in Figure 2. The high fever, allergy, and nausea/vomiting incidence rates between Ctrl and three dose groups demonstrated no considerable differences (*P*>0.05). The overall incidence rates of ARs were 13.33%, 13.33%, 6.67%, and 10.00% in Ctrl and three dose groups, respectively. Nevertheless, the overall incidence rates of ARs differed slightly between Ctrl and three dose groups (*P*>0.05).

## Discussion

VP is a respiratory disease caused by viral infection, characterised by symptoms such as high fever, cough, dyspnea, and chest pain. Some patients may also experience fatigue, malaise, and other systemic symptoms [13]. Common clinical approaches involve antipyretics, cough suppressants, expectorants, and antiviral medications to alleviate symptoms. MPS belongs to the class of corticosteroid medications and can inhibit the infiltration of inflammatory cells at the site of inflammation, thus relieving local inflammatory reactions [14]. By reducing pulmonary inflammation and swelling, MPS can improve lung function and help alleviate respiratory distress symptoms in patients [15]. MPS suppresses immune system responses and lymphocyte activity, thereby reducing inflammation and immune reactions, exerting immunosuppressive effects [16]. Some studies indicated that MPS can alleviate allergic reactions and have certain effects on physiological processes such as protein synthesis, carbohydrate metabolism, and lipid metabolism [17]. Currently, the adoption of MPS in treating VP remains controversial. Some studies suggest that glucocorticoids may be beneficial in severe pulmonary inflammation and the release of inflammatory factors [18] [19].

Nevertheless, other research suggested that in certain circumstances, excessive use of steroid medications may lead to immunosuppression and increase the risk of bacterial infections and other side effects [20]. Azithromycin is a semi-synthetic macrolide antibiotic that, when absorbed by the body quickly, can effectively kill bacteria and viruses, thereby achieving the therapeutic goal. Recent research findings noted that the combination therapy of MPS and azithromycin has better efficacy in treating Mycoplasma pneumoniae pneumonia, significantly reducing recovery time and attenuating the severity of inflammatory reactions in patients undergoing combinationtherapy [21]. The TER post-treatment in the Ctrl group was 60.00%, which was inferior to those in the three dose groups, 76.67%, 90.00%, and 93.33%, respectively (*P*<0.05). Furthermore, the TER in the L-MPS group was inferior to that in other dose groups (*P*<0.05). It can be observed that MPS exhibits different therapeutic effects at different doses when treating patients with VP. The therapeutic effects in the medium-dose and high-dose groups may be comparable, while the therapeutic effect in the low-dose group is relatively poor. Nevertheless, all doses showed an increase in the effective rate compared to the control group, indicating that MPS combined with azithromycin has a specific therapeutic effect in treating patients with VP. There may be a dose-dependent relationship when using MPS to treat VP.

CRP and PCT are two commonly used biochemical markers for assessing the body’s inflammation and infection status. They indicate the body’s inflammatory response, with concentrations significantly increasing when infected or inflamed [22]. In VP, the elevation of these two markers reflects the severity of the inflammatory process, aiding in the assessment of inflammation severity. PCT shows a rapid increase in the early stages of infection, making it crucial to diagnose VP early. CRP and PCT levels gradually decrease as treatment progresses, indicating disease control and improvement. It was revealed that post-treatment, CRP and PCT decreased in all groups (*P*<0.05).

Additionally, post-treatment CRP and PCT in three dose groups were inferior to those in the Ctrl group (*P*<0.05), while CRP and PCT in the L-MPS group were superior to those in other dose groups (*P*<0.05). Different concentrations of MPS combined with azithromycin treatment have a marked reducing effect on inflammatory markers (CRP and PCT) in patients with VP, indicating its good control over inflammatory responses. There are differences in the impact of MPS at different doses on inflammatory markers in patients with VP, with higher and moderate doses showing better efficacy than lower doses. MPS exhibits significant anti-inflammatory and anti-allergic effects by inhibiting the release of inflammatory mediators, thereby alleviating inflammatory reactions [23]. On the one hand, azithromycin exerts antibacterial and anti-inflammatory effects by inhibiting protein synthesis and affecting cellular signalling pathways [24]. The combination of the two may produce a synergistic effect, enhancing the anti-inflammatory action and consequently decreasing serum CRP and PCT levels.

On the other hand, the combined use of MPS and azithromycin can simultaneously target inflammation and potential bacterial infections, thereby alleviating the inflammatory response caused by pathogens and reducing serum CRP and PCT levels. MPS exhibits anti-inflammatory effects and aids in tissue repair and regeneration, reducing the release of inflammatory mediators [25]. This helps alleviate the inflammatory response caused by lung tissue damage, leading to a decrease in serum CRP and PCT levels.

IL-6 is a multifunctional cytokine in inflammation and immune responses. In VP, IL-6 production increases, promoting the occurrence of inflammatory responses and cytokine release, which helps regulate immune responses [26]. IL-6 can also induce fever by affecting the central temperature regulation centre, aiding the body in responding to pathogen infections. Additionally, IL-6 participates in lung tissue repair processes, regulating cell proliferation and repair, thereby playing an essential role in restoring lung function [27]. TNF-α is a pro-inflammatory cytokine that mediates inflammatory responses during inflammation. In VP, the release of TNF-α leads to the infiltration of inflammatory cells and the release of inflammatory mediators, exacerbating the inflammatory response. Excessive TNF-α production may cause inflammation-induced lung tissue damage, further impairing lung function [28]. TNF-α also influences the activation and regulation of immune cells, participating in the body’s immune response processes [29]. In summary, the roles of IL-6 and TNF-α in VP mainly involve regulating inflammatory responses, promoting lung tissue repair, and influencing immune responses. Proper regulation of the levels of these two inflammatory mediators helps control the inflammatory response caused by VP, alleviates symptoms, and promotes recovery.

The results presented in this study show that compared to pre-treatment, serum CRP and PCT levels decreased markedly post-treatment in all groups (*P*<0.05). Additionally, IL-6 and TNF-α levels post-treatment were lower in three dose groups versus the Ctrl group (P<0.05), while they were higher in the L-MPS group compared to other dose groups (*P*<0.05). It can be observed that the serum CRP and PCT levels of patients in all four groups decreased drastically post-treatment versus pre-treatment, indicating a notable regulatory effect of treatment on the inflammatory levels. This suggests that the combined treatment of MPS and azithromycin significantly impacts the inflammatory indicators of patients with VP. Post-treatment, IL-6 and TNF-α in three dose groups were notably inferior to those in the Ctrl group, indicating that combined treatment with different doses of MPS and azithromycin can effectively reduce inflammatory mediators. The research results also showed that IL-6 and TNF-α in the L-MPS group were superior to those in other dose groups, suggesting that lower doses of MPS may not be as effective in anti-inflammatory action as medium and high doses. The combined treatment of MPS and azithromycin can significantly reduce the levels of inflammatory indicators (CRP and PCT) and regulate the secretion of inflammatory mediators in patients with VP. Furthermore, according to the research findings, medium and high doses of MPS may be more effective in reducing the levels of inflammation in patients with VP compared to low doses.

The duration of fever, cough, and X-ray infiltration disappearance in three dose groups of VP patients was shorter than the Ctrl group (*P*<0.05). This suggests that combined treatment with different doses of MPS and azithromycin can accelerate symptom relief and improve X-ray manifestations in patients with VP, thereby helping to shorten the duration of the disease and improve recovery speed. The duration of fever, cough, and X-ray infiltration disappearance in the L-MPS group was longer than that in other dose groups (*P*<0.05), indicating that the effectiveness of low-dose MPS in relieving symptoms and improving X-ray manifestations in patients with VP was inferior to that of medium and high doses of MPS. This may be directly related to the dosage, as the control effect of lower doses on the condition may be affected to some extent. The lung function indicators FVC, MMEF, and PEF in three dose groups of VP patients were superior to those in the Ctrl group (*P*<0.05), indicating that combined treatment with different doses of MPS and azithromycin can significantly improve lung function in patients. This treatment enhances the values of FVC, MMEF, and PEF, contributing to restoring respiratory function in patients. The FVC, MMEF, and PEF values in the L-MPS group were relatively higher (*P*<0.05), implying that moderate and high doses of MPS combined with azithromycin on improving lung function may be more significant than those of low doses. Higher doses of MPS combined with azithromycin may have a more substantial effect on enhancing patients’ lung function and respiratory capacity. According to the results, the total incidence of ARs demonstrated neglectable differences among the groups (*P*>0.05). This suggests a slight difference in the total incidence of ARs among patients treated with low, moderate, or high doses of MPS combined with azithromycin.

To ensure the safety of drug therapy, selecting the appropriate dosage is crucial in clinical practice. It is essential to adhere to the principles of early drug administration, using small doses and minimising the duration of treatment. The results indicated that a moderate dose (80 mg) of MPS combined with azithromycin could greatly reduce inflammatory cytokine levels in patients with VP, shorten the time to symptom relief, improve lung function and respiratory capacity, and demonstrate significant efficacy in treating VP. Therefore, it can be considered the preferred dosage for clinical treatment of VP.

## Conclusion

This study investigated the effects of different doses of MPS combined with azithromycin on serum biochemical factors and prognosis in patients with VP. The results indicated that a moderate dose (80 mg) of MPS combined with azithromycin significantly improves serum biochemical factor levels, shortens the time to symptom relief, and enhances lung function in patients with VP. Nevertheless, the limited sample size precludes definitive conclusions regarding the long-term efficacy and safety of the treatment approach. Future work will focus on expanding the sample size to validate the results. In conclusion, this study provides valuable insights for developing treatment strategies for VP.

## Dadatak

### Acknowledgements

We would like to express our sincere gratitude to all the patients who participated in this study and the medical staff of the Department of Traditional Chinese Medicine, Zigong First People’s Hospital, for their support and cooperation.

### Funding

The research is supported by: 1 This research was supported by Sichuan Provincial; Administration of Traditional Chinese Medicine (No.: 2021MS462); 2 This research was supported by Sichuan Zigong City Science and Technology Bureau (No.: 2021YXY01); 3 This research was supported by Sichuan Zigong Municipal Health Commission (No.: 2019yb009).

### Ethical consideration

This study was approved by the Ethics Committee of Zigong First People’s Hospital (approval number: [approval number]). The study was conducted following the principles of the Declaration of Helsinki and the International Conference on Harmonization Good Clinical Practice (ICH-GCP) guidelines. Written informed consent was obtained from all patients before they participated in the study.

### Author contributions

Hongping Zeng: Concept and design of the study, data collection, and manuscript writing.

Qi Zhu: Data collection, data analysis, and manuscript revision.

Shaoyu Bai: Patient recruitment, data collection, and manuscript revision.

Jie Liu: Data analysis and manuscript revision.

Dengjie Ren: Data collection and manuscript revision.

Xin Chen: Concept and design of the study, data analysis, manuscript writing, and final approval of the manuscript.

### Conflict of interest statement

All the authors declare that they have no conflict of interest in this work.
